# Some Scientific Aspects of Insanity

**Published:** 1892-02-27

**Authors:** 


### Some Scientific Aspects of Insanity.

### XIV.—SECONDARY DEMENTIA.

Like primary dementia, it is characterised by mental weakness, but it differs from it in being accompanied by a loss of memory, of attention, of curiosity, of interest in surroundings, and by a want of energy and originality in a person who at one period of his life possessed these qualities. The patients belonging to this class make up the large majority of the chronic inmates of modern lunatic asylums. When one enters one of the large chronic wards of an English county asylum, a ward which contains from fifty to one hundred and fifty of those mindless, thoughtless, hopeless cases, and when one remembers that most of them have at some period of their existence enjoyed the use of their mental faculties, the thought that in the midst of life we are surrounded by death must forcibly occur. Such a ward is a living sepulchre, a vast mindless wilderness of human vegetative life, the great majority of the inmates of which, were they to be stranded on a desert island, even though that island contained all the raw materials necessary for human food, would collectively starve to death; or were they individually turned adrift into the streets of a populous city, they would die for lack of the necessary originality to procure a livelihood. Such is their lack of interest in their surroundings that murders and suicides have been known to take place in asylums in presence of demented patients without exciting the smallest degree of interest, or disturbing their apathy. Those who have been long in connection with the insane know how little curiosity is exhibited by this class of asylum inmates in the arrival of new doctors or attendants, or in the fate of their fellow patients or officials who have left the institution. This dementia may be secondary (*a*) to previous attacks of insanity when it is known simply as secondary dementia. (*b*) To prolonged and deep indulgence in alcohol, when it is called alcoholic dementia. (*c*) To gross lesions of the brain, causing paralysis, when it is known as paralytic dementia. (*d*) To the tear and wear of a long and hard life, when it is known as senile dementia.

Secondary Dementia proper follows upon previous attacks of acute or chronic insanity. It is, as I have already quoted from the writings of a well-known alienist, the "goal of all insanities." It may follow a sharp attack of mania, some forms of melancholia, mono-mania, and epileptic insanity. Some have even regarded those acute attacks as stages in the course of the disease dementia, and there can be no doubt that it supervenes most commonly upon that form of mania which attacks persons at the adolescent period of life. It is the most typical form of dementia, and that most commonly met with among large masses of the chronic insane.

Alcoholic dementia is a distinct form of secondary dementia. It generally occurs in those people who have persistently soaked in alcohol for years. It is not so apt to occur in those who have periodic drinking bouts, or who may be the subjects of occasional attacks of delirium tremens. It is the steady drinker, who may never get absolutely drunk, but who nevertheless drinks persistently from year's end to year's end, that is most liable to this form of insanity. The striking peculiarity of alcoholic dementia is the complete failure of memory for recent events. The patient may remember with accuracy and discuss fluently historical questions requiring a knowledge of dates, events in his past history, even his present position, but in the great majority of cases within twelve hours, often within five minutes, he will have totally forgotten that he held such a conversation with a second person.

